# L‐glutamine protects mouse brain from ischemic injury via up‐regulating heat shock protein 70

**DOI:** 10.1111/cns.13184

**Published:** 2019-06-20

**Authors:** Long‐Long Luo, Yong‐Fang Li, Hui‐Min Shan, Li‐Ping Wang, Fang Yuan, Yuan‐Yuan Ma, Wan‐Lu Li, Ting‐Ting He, Yu‐Yang Wang, Mei‐Jie Qu, Huai‐Bin Liang, Zhi‐Jun Zhang, Guo‐Yuan Yang, Yao‐Hui Tang, Yong‐Ting Wang

**Affiliations:** ^1^ Shanghai Jiao Tong Affiliated Sixth People’s Hospital, Neuroscience and Neuroengineering Research Center, Med-X Research Institute and School of Biomedical Engineering Shanghai Jiao Tong University Shanghai China; ^2^ Department of Neurology, School of Medicine, Ruijin Hospital Shanghai Jiao Tong University Shanghai China; ^3^ Department of Rehabilitation Medicine, The Affiliated Hospital of Qingdao University Qingdao University Qingdao China; ^4^ Department of Neurology, The Affiliated Hospital of Qingdao University Qingdao University Qingdao China

**Keywords:** heat‐shock protein 70, ischemic stroke, L‐glutamine, neuroprotection, oxidative stress

## Abstract

**Introduction:**

L‐glutamine is an antioxidant that plays a role in a variety of biochemical processes. Given that oxidative stress is a key component of stroke pathology, the potential of L‐glutamine in the treatment of ischemic stroke is worth exploring.

**Aims:**

In this study, we investigated the effect and mechanisms of action of L‐glutamine after cerebral ischemic injury.

**Results:**

L‐glutamine reduced brain infarct volume and promoted neurobehavioral recovery in mice. L‐glutamine administration increased the expression of heat‐shock protein 70 (HSP70) in astrocytes and endothelial cells. Such effects were abolished by the coadministration of Apoptozole, an inhibitor of the ATPase activity of HSP70. L‐glutamine also reduced oxidative stress and neuronal apoptosis, and increased the level of superoxide dismutase, glutathione, and brain‐derived neurotrophic factor. Cotreatment with Apoptozole abolished these effects. Cell culture study further revealed that the conditioned medium from astrocytes cultured with L‐glutamine reduced the apoptosis of neurons after oxygen‐glucose deprivation.

**Conclusion:**

L‐glutamine attenuated ischemic brain injury and promoted functional recovery via HSP70, suggesting its potential in ischemic stroke therapy.

## 1 INTRODUCTION

Stroke is the second leading cause of death and one of the leading causes of disability worldwide.^1^, ^2^ Among all possible pathological processes occurring after ischemic stroke, free radical damage and oxidative stress have been found to play a key role in stroke.^3^, ^4^ There is an increasing amount of experimental evidence that oxidative stress is a causal, or at least an ancillary factor in the neuropathology of stroke. The mechanism of oxidative stress‐induced neuronal death in ischemic stroke has been extensively studied.^5^, ^6^ It is now well established that different molecular changes converge during brain ischemia and reperfusion to produce damaging concentrations of reactive oxygen species (ROS) and reactive nitrogen species (RNS) that can prevent clinical improvement, which promotes lipid peroxidation, mitochondrial and DNA damage, protein nitration and oxidation, depletion of antioxidant reserves, activation or inhibition of multiple signaling pathways, and breakdown of the blood‐brain barrier.^7^, ^8^

L‐glutamine is an antioxidant that was approved by the Food and Drug Administration in 2017 for the treatment of sickle cell anemia.^9^ In the enzymatic antioxidant system, glutathione (GSH) and superoxide dismutase (SOD) are the most important antioxidants which work together to counteract oxidative stress in cells and protect brain from ischemia‐reperfusion damage. L‐glutamine is a precursor of reduced GSH, which had been shown to have antioxidative stress effects. Therefore, we tried to study whether direct supplementation of L‐glutamine can provide the same oxidative stress protection on brain injury.

L‐glutamine is involved in nitrogen transport, regulation of acid‐base homeostasis, and catabolic signaling.^9^ It is also a substrate for glutathione synthesis, basic building block for proteins, and a potential inhibitory agent for inflammatory cytokine release.^10^ The glutamate‐glutamine cycle is thought to be integral in continuously replenishing the neurotransmitter pool of glutamate. Neurotransmitter glutamate is released from the presynaptic terminals of neuron and interacts with receptors in the postsynaptic membrane. After uptake into astrocytes, glutamate is converted to glutamine by glutamine synthetase which is exclusively expressed in glial cells.^11^ Ischemia results in ATP loss, which contributes to the paralysis of glutamate transporters that normally remove released glutamate from the synaptic cleft; the excess of glutamate in extracellular space leads to excessive activation of glutamate receptors and pathological rise of Ca2^+^; neuron is consequently subjected to overwhelming ion flux, leading to the occurrence of excitotoxicity.^12^ Studies have shown that increasing the net glutamine output in the glutamate‐glutamine cycle after brain injury reduced glutamate excitotoxicity, and protected neuronal viability.^13^ The application of 0.75 g/kg dipeptide alanyl glutamine, as an effective L‐glutamine supplement, increased plasma glutamine without elevating brain glutamate in patients, which indicated that appropriate L‐glutamine administration was not associated with signs of potential glutamate‐mediated cerebral injury.^14^

Heat‐shock proteins (HSPs) are induced by various of environmental stresses and classified into several families on the basis of their apparent molecular sizes, including HSP110, HSP90, HSP70, HSP60, HSP32, and small HSPs.^15^, ^16^, ^17^ Several studies have shown that HSPs are involved in protecting brain from ischemic stroke which could be attributed to their chaperone functions.^18^, ^19^ Among all the HSPs, HSP70 is a central component in the cellular network of molecular chaperones and folding catalysts as well as a highly stress‐inducible member of a chaperone protein family.^20^ Studies suggest that L‐glutamine enhances HSP70 expression through the hexosamine biosynthetic pathway (HBP) where the amino group from glutamine is transferred to fructose‐6‐phosphate by glutamine fructose‐6‐amidotransferase (GFAT) and eventually yield UDP‐N‐acetyl glucosamine (UDP‐GlcNAc). Studies using knockout models have clarified that the induction of HSP is necessary for the beneficial effects of L‐glutamine supplementation after injury.^21^

It has been reported that HSP70 induced by L‐glutamine protected against ischemia‐reperfusion injury in different organs including kidney, intestine, liver, and lung,^22^, ^23^ however, it remains unclear whether L‐glutamine treatment can protect against cerebral ischemic injury. In this study, we investigated the effect of L‐glutamine (L‐GLN), a recent FDA‐approved antioxidant, in attenuating ischemic brain injury. Our work explored the potential interventions of cerebral ischemic injury using this clinically approved molecule. We believe this study opens new possibilities in stroke treatment using L‐glutamine.

## 2 MATERIALS AND METHODS

### 2.1 Experimental design

All animal protocol was approved by the Institutional Animal Care and Use Committee of Shanghai Jiao Tong University, China (Permission number: Bioethics 2012022), and appropriate measures were taken to ensure minimal pain or discomfort of the animals. Reporting of these experiments complies with the ARRIVE (Animal Research: Reporting in Vivo Experiments) guidelines. A 8‐week‐old male ICR mice were used in the experiments. 90‐minute middle cerebral artery occlusion (MCAO) surgery was performed. A total of 208 mice were used for the experiment with a mortality rate of 23.1%. A 2.4% of mice with no hemiplegia symptoms or apparent brain infarction after surgery were excluded from further analysis. The final allocation of the 155 animals in the five experimental groups is as follows: (a) Sham (n = 10), (b) Saline treated (n = 37), (c) L‐glutamine treated (n = 48), (d) L‐glutamine plus Apoptozole treated (n = 35), and (e) Apoptozole alone treated (n = 25).

The oxygen‐glucose deprivation (OGD) experiment was performed in three groups: (a) Astrocytes or BEND.3 cells cultured with L‐glutamine‐free medium; (b) Astrocytes or BEND.3 cells cultured using L‐glutamine supplemented medium; (c) Astrocytes or BEND.3 cells cultured using medium added with L‐glutamine and Apoptozole. Astrocytes or BEND.3 cells alone cultured with L‐glutamine‐free medium and without OGD were used as control. Subsequently, neurons cultured with four corresponding conditioned mediums after OGD were used for further analysis.

### 2.2 MCAO surgery

Animals were anesthetized with 1.5% isoflurane in a 30% O2/68.5%NO mixture under spontaneous breathing conditions. A 6‐0 suture (Covidien) coated with silicon was gently inserted from the external carotid artery (ECA) with an advancement of 9‐10 mm until reaching the intersection of the middle cerebral artery (MCA). After 90‐minute ischemia, the suture was withdrawn. A laser Doppler flowmetry (Moor Instruments) was used to monitor the blood flow in the MCA territory before surgery, immediately after occlusion, and reperfusion. Successful occlusion of MCA was confirmed as a decline in the regional blood flow of ipsilateral hemisphere by more than 80% compared to the contralateral hemisphere.

### 2.3 Drug administration

For the therapeutic window study, 0.75 g/kg L‐glutamine (Thermo Fisher Scientific) or vehicle (0.9% saline) was given intraperitoneally at 0 hours after MCAO on the first day and then once daily for a total of 3 days. In order to ensure the inhibitory effect of HSP70 activity in vivo and protect mice from potential toxicity, we administered the doses of Apoptozole (AZ, MedChem Express) as reported in the previous literature (4 mg/kg/d). Apoptozole stock solution (10 mmol/L in DMSO, MedChem Express) was diluted in 1× PBS buffer with a final DMSO concentration of 2%, ±0.01% v/v Triton X‐100 (addition of the nonionic detergent Triton X‐100 prevented the formation of large aggregates) for the inhibitor group. The first dose was given immediately after MCAO, and then, the second dose was given 48 hours after MCAO.^24^ To further explore dose response in astrocytes and BEND.3 cells, a range of L‐glutamine (0, 1, 2, 3, 4, 5, and 6 mmol/L) or Apoptozole (0, 5, 10, 15, 20, and 25 μmol/L) was given immediately after OGD for 24 hours.

### 2.4 Neurobehavioral assessments

Neurobehavioral tests were performed before MCAO and at 1, 3, 7, and 14 days after MCAO by an investigator blinded to the experimental design using the modified neurological severity score (mNSS), elevated body swing test (EBST), hanging wire test, and rotarod test (Rotor‐Rod). The detailed criteria are shown in Table S1.

### 2.5 Infarct volume measurement

A total of 72 hours after stroke, the mouse was perfused with 0.9% saline and fixation with 4% paraformaldehyde (Sinopharm Chemical Reagent), and mouse brain was cut into 20‐μm‐thick brain sections by a microtome. Cryosections (200 μm apart) were obtained from the brain, and the infarct volume was measured by 0.05% Cresyl Violet acetate (Sigma‐Aldrich) staining. The contralateral area minus the normal area of the ipsilateral hemisphere was recorded as the infarct area ΔS. The infarction area of two adjacent pieces is denoted as ΔS1 and ΔS2; the volume of infarction (V) for two adjacent cerebral volumes is H/3 × [ΔS1 + (ΔS1 × ΔS2)^1/2^ + ΔS2]; the thickness (H) = 0.2 mm. Then, cerebral infarction volumes between two adjacent brain tissues were added to yield the total infarction volume.^2^

### 2.6 Immunostaining and quantification

Brain sections or cells were blocked for 60 minutes in 10% bovine serum at room temperature albumin after treatment with 4% PFA and 0.3% Triton X‐100 for 10 minutes sequentially, thereby the samples were incubated with primary antibodies against HSP70, Ki67 (Abcam), GFAP (Millipore), NeuN (Millipore), MAP2(Millipore), CD31 (R&D Systems), and IBA‐1 (WAKO) overnight at 4°C. After washing with PBS, sections or cells were incubated with secondary antibody for 1 hour at 37°C and DAPI (Beyotime Biotechnology). For apoptosis analysis, TUNEL staining was performed by using an in situ Cell Death Detection Kit (Roche Diagnostics). Five fields were sampled for each brain sections, and four brain sections were assessed for each animal. The number of TUNEL‐positive cells was quantified using ImageJ software (NIH).

### 2.7 Western blotting analysis

Of 24 and 72 hours after stroke, proteins were extracted from the ipsilateral hemisphere of cortex and striatum of the mouse and placed in RIPA Lysate (Millipore). The Western blot protocol was performed as previously described, and the primary antibodies were HSP70 (Abcam), GFAP (Millipore), Nuclear factor erythroid‐2‐related factor 2 (Nrf2, Santa Cruz Biotechnology), Brain‐derived neurotrophic factor (BDNF, Santa Cruz Biotechnology), BCL2‐associated X protein (BAX, Abcam), B‐cell lymphoma 2 (BCL2, Cell Signaling Technology), Nuclear factor kappa‐B (NF‐κB p‐P65/p65, Cell Signaling Technology), and Signal transducer and activator of transcription 3 (STAT3, Cell Signaling Technology). β‐actin (Santa Cruz Biotechnology) was employed as the loading control. Immunoblots were detected using an enhanced chemiluminescence kit (FD Technology) and calculated using ImageJ software (NIH).

### 2.8 Real‐time polymerase chain reaction analysis

The total RNA from the ipsilateral hemisphere was extracted from tissues around the lesion sites at 24 and 72 hours after MCAO using TRIzol (Life Technologies), and real‐time PCR was performed according to the manufacturer's instructions. The cDNA was synthesized by reverse transcriptase‐polymerase chain reaction (PCR) using a SYBR Premix Ex Taq Kit (Life Technologies). All procedures were performed following the manufacturer's protocol. Gene transcription was detected by real‐time PCR in a 7900HT sequence detection system (Applied Biosystems) using specific primers designed from known sequences. GAPDH (Cell Signaling Technology) was used as an endogenous control. Sequence‐specific primers for HSP10, HSP27, HSP32, HSP60, HSP70, HSP90, HSP110, IL‐1β, IL‐6, TGF‐β, IL‐10, and GADPH are shown in Table S2.

### 2.9 Oxidative stress analysis

The supernatants of serum from animals were subjected to SOD, GSH, and MDA assays using commercial kit (Jiancheng Bioengineering Institute) to assess the antioxidative ability of the tissue.

For detection of reactive oxygen species (ROS) in vitro, astrocytes were seeded on 6‐well plates, exposed to OGD for 5 hours, and then treated with 2 mmol/L L‐glutamine medium or L‐glutamine‐free medium for 24 hours. For the inhibitor group, astrocytes were treated with 2 mmol/L L‐glutamine + 15 μmol/L Apoptozole for 24 hours. After treatment, astrocytes were incubated with 10 μmol/L DCFH‐DA (Beyotime, Shanghai, China) for 20 minutes at 37°C. Oxidation of DCFH by ROS produces the highly fluorescent DCF, which was monitored at 488 nm (excitation)/525 nm (emission) by a confocal laser‐scanning microscope (Leica). The fluorescence intensity of the cells was measured and analyzed by flow cytometry using FACScan (Beckman coulter cell).

### 2.10 Cells culture and drug treatment

BEND.3 cell line was purchased from American Type Culture Collection. Primary astrocytes were prepared from newborn ICR mice (within 24 hours after birth). BEND.3 cells and astrocytes were cultured in Dulbecco's modified Eagle medium (DMEM, containing 4 mmol/L L‐glutamine) supplemented with 10% fetal bovine serum (FBS; GIBCO). The culture medium was renewed every three days. For the following experiments, BEND.3 cell and the pure secondary astrocytes were then cultured in L‐glutamine‐free medium or 2 mmol/L L‐glutamine or 2 mmol/L L‐glutamine + 15 μmol/L Apoptozole for 24 hours after 5 hours of OGD and blank group was cultured in L‐glutamine‐free medium without OGD.

Primary neurons were obtained from embryos of pregnant ICR mice (16 days). Cells were seeded on Poly‐D‐lysine (Sigma‐Aldrich) coated culture flasks and grown for 4 hours in DMEM before changing into Neurobasal (Gibco) containing 2% B27 (Gibco) and 0.5 mmol/L L‐glutamine. The culture medium was half‐renewed every three days, and mature neurons were obtained from 6th day to 10th day. The mature neurons were subjected to OGD for 1 hour to induce cell injury and consequently treated the injured neurons with 1:1 mixture of fresh complete Neurobasal without L‐glutamine and CM from 0 or 2 mmol/L L‐glutamine or 2 mmol/L L‐glutamine + 15 μmol/L Apoptozole‐treated OGD astrocytes or OGD‐BEND.3 for 24 hours.

### 2.11 Cell viability and cytotoxicity assessment

Cell viability was evaluated using Cell Counting Kit‐8 (CCK‐8; Beyotime). The absorbance at 450 nm was read using a microplate reader (BioTek). The supernatant of the OGD cells was collected for cytotoxicity assessment using a lactate dehydrogenase (LDH) kit (Beyotime). Samples were measured for absorbance at 490 nm with a microplate reader (BioTek).

### 2.12 Statistical analysis

The parametric data were analyzed using SPSS v24.0 (SPSS Inc). One‐way ANOVA followed by Bonferroni post hoc tests was used for statistical comparisons among multiple groups. All data were expressed as mean ± SD, and *P* < 0.05 was considered statistical significance.

## 3 RESULTS

### 3.1 L‐glutamine treatment reduced infarct volume and promoted neurobehavioral recovery in mice after stroke

**Figure 1:**
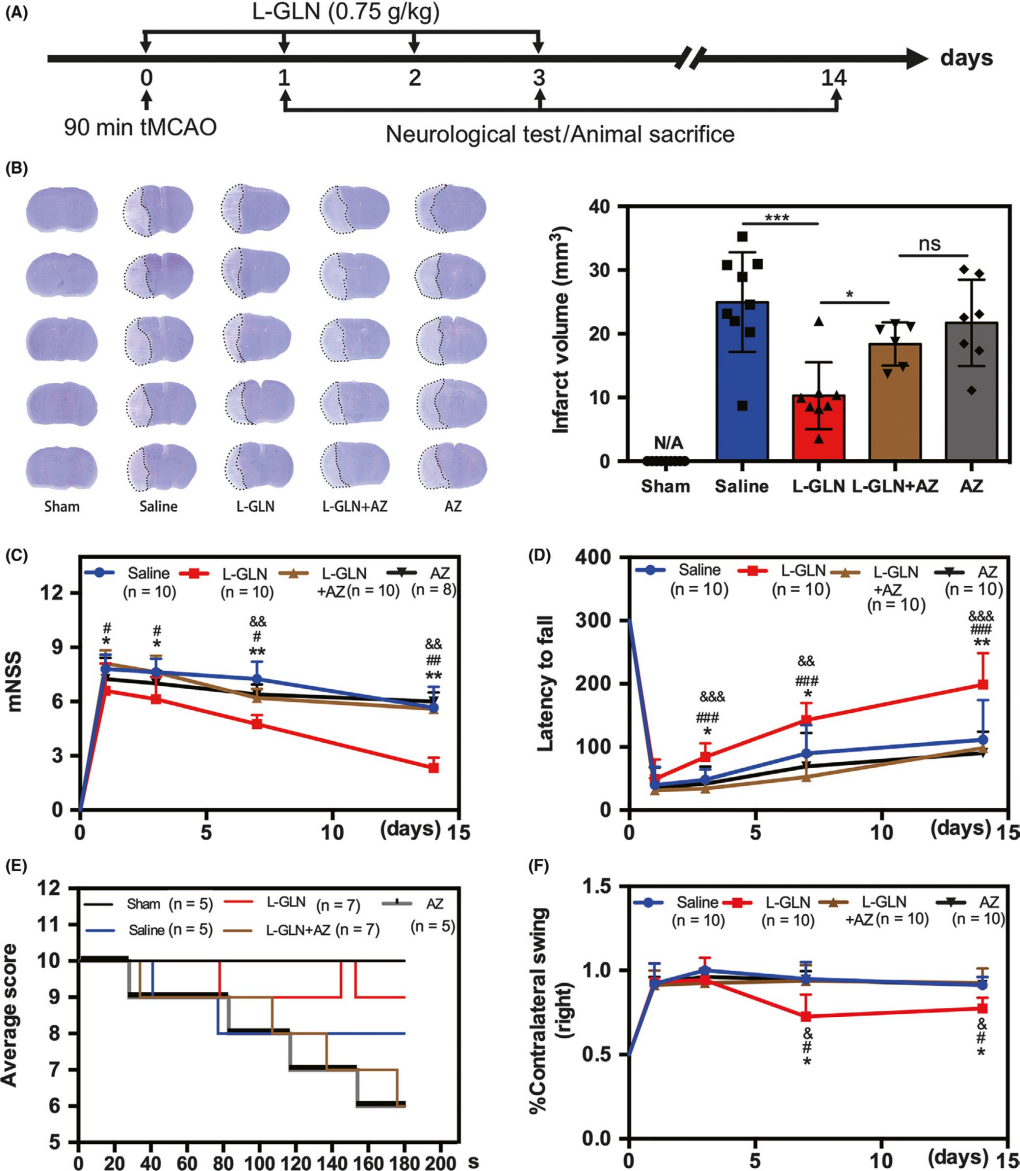
L‐glutamine reduced brain infarct volume and promoted neurobehavioral recovery in mice. A, The experimental scheme. B, Representative cresyl violet‐stained brain sections at 72 h and the statistics of infarct volume of sham (n = 9), saline (n = 9), L‐glutamine (n = 8), L‐glutamine + Apoptozole group (n = 6), and Apoptozole alone group (n = 7), *P < 0.05, ***P < 0.001. Line graphs showed four separate behavioral tests including modified neurological severity score (C), rotarod performance tests (D), hanging wire tests (E), and elevated body swing tests (F), n = 5‐10 per group, from C‐F, *P < 0.05, **P < 0.01 (L‐glutamine group vs. saline group), ^#^ *P < *0.05, ^##^ *P < *0.01, ^###^ *P < *0.001(L‐glutamine group vs. L‐glutamine + Apoptozole group). ^&^ *P < *0.05, ^&&^ *P < *0.01, ^&&&^ *P < *0.001(L‐glutamine group vs. Apoptozole alone group). Data are presented as mean ± SD

Our experiment was designed as illustrated in Figure 1A. Laser speckle contrast imaging was used to monitor blood flow in brain cortex after suture occlusion and reperfusion (Figure S1A). L‐glutamine injection significantly reduced infarct volume, while addition of Apoptozole reversed the protective effect (Figure 1B). Of 4 mg/kg Apoptozole‐only treatment did not affect the infarct volume of mice. The mNSS score of mice in the L‐glutamine‐treated group was lower than that of the saline‐treated group, the L‐glutamine + Apoptozole‐treated group, and the Apoptozole alone group at 1, 3, 7, and 14 days after MCAO (Figure 1C). Treatment with L‐glutamine also improved animal performance in rotarod test at 3, 7, and 14 days (Figure 1D), hanging wire tests at 14 days (Figure 1E), and EBST at 7 and 14 days after MCAO (Figure 1F). In addition, our results showed that there was no difference in behavioral performance of stroke mice among Apoptozole alone group, L‐glutamine + Apoptozole group, and saline group.

### 3.2 L‐glutamine treatment upregulated HSP70 expression, increased antioxidant levels, and reduced inflammatory response in stroke mice

**Figure 2:**
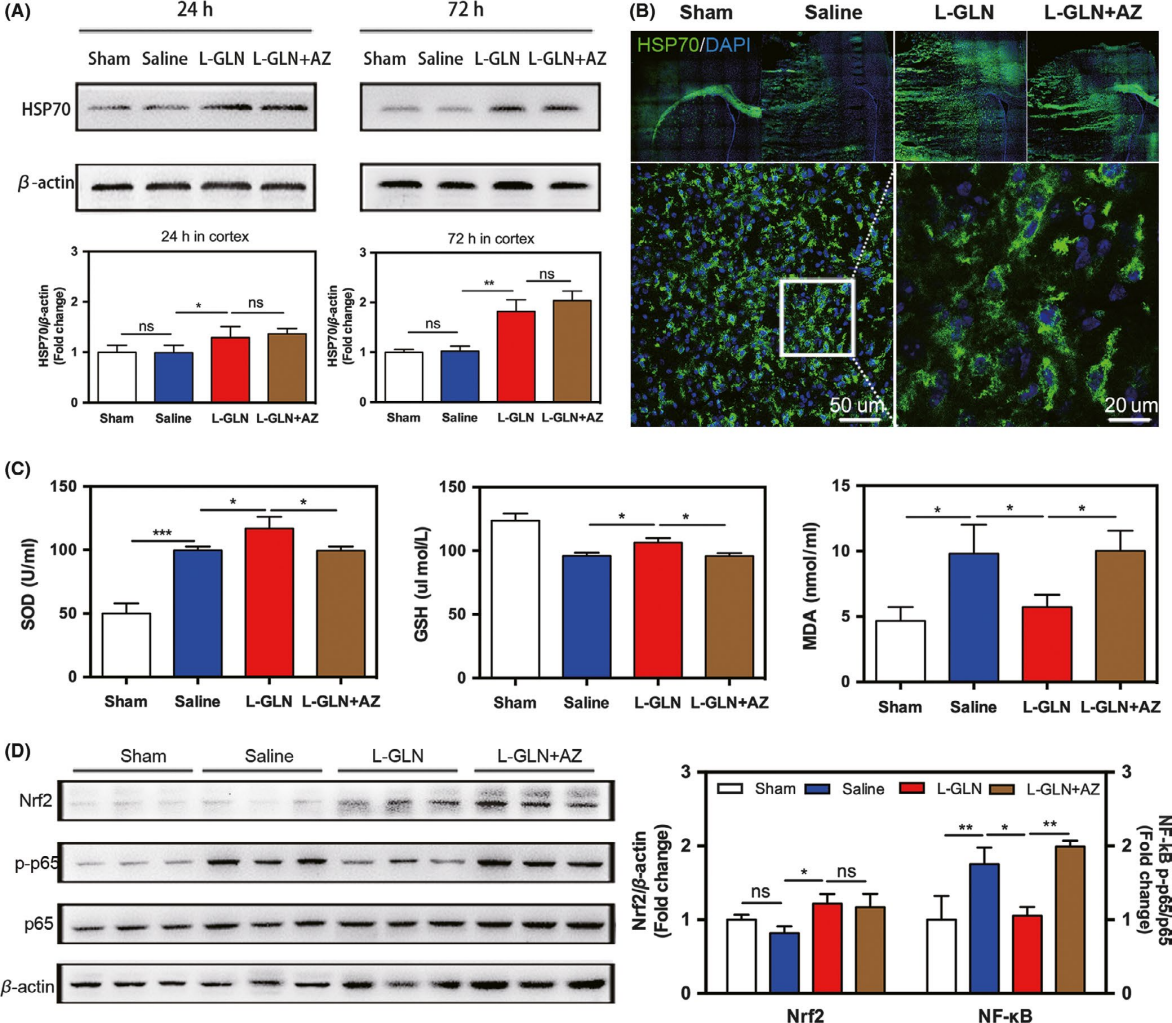
L‐glutamine treatment upregulated HSP70, increased antioxidant levels, and reduced inflammatory response in stroke mice. A, Western blot analysis of HSP70 from the 4 groups at 24 and 72 hours in cortex. B, Immunostaining of HSP70 (green) and DAPI (violet) in sham, saline, L‐glutamine, and L‐glutamine + Apoptozole group. Bar = 50 μm/20 μm. C, Bar graph showed SOD, GSH activity, and MDA level of the 4 groups. D, Western blot analysis of Nrf2 and NF‐κB (p65) expression at 24 hours of the 4 groups. β‐actin or p65 was used as loading control, respectively. N = 5‐6 per group. Data are presented as mean ± SD, *P < 0.05, **P < 0.01, ***P < 0.001

Western blot confirmed that L‐glutamine treatment increased HSP70 expression in the cortex at 24 hours and 72 hours after MCAO (Figure 2A), while no significant difference in the striatum until 72 hours (Figure S2B). It is known that Apoptozole inhibits the ATPase activity of HSP70 by binding to its ATPase domain but not affects HSP70 expression.^24^ Indeed, we found Apoptozole treatment did not affect HSP70 upregulation caused by L‐glutamine treatment (Figure 2B). Oxidative stress assessment indicated that L‐glutamine increased SOD and GSH levels and decreased MDA level compared with the saline group (Figure 2C). Western blot revealed that L‐glutamine treatment increased the expression of Nrf2 and decreased that of NF‐κB at 24 hours after ischemic stroke (Figure 2D).

### 3.3 L‐glutamine increased HSP70 expression in astrocytes and endothelial cells and reduced neuron apoptosis in stroke mice brain

**Figure 3:**
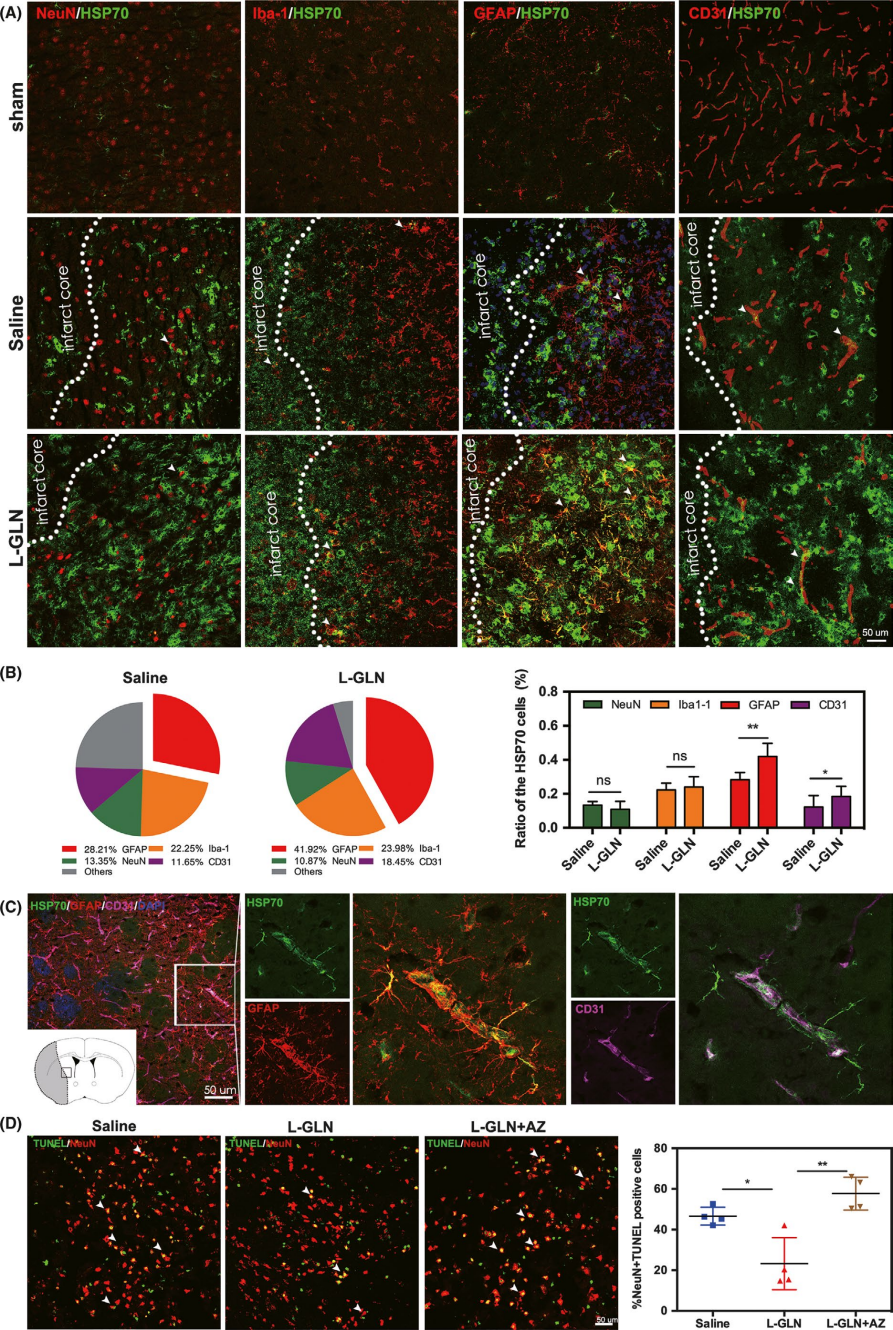
L‐glutamine increased HSP70 in astrocytes and endothelial cells of stroke mouse brain and reduced neuronal apoptosis. A, Double immunofluorescent staining of HSP70 (green)/NeuN (red), HSP70 (green)/Iba‐1 (red), HSP70 (green)/GFAP (red), and HSP70 (green)/CD31 (red) in the saline and L‐glutamine groups. Bar = 50 μm. B, Statistical analysis of HSP70^+^/NeuN^+^, HSP70^+^/Iba‐1^+^, HSP70^+^/GFAP^+^, and HSP70^+^/CD31^+^ cells in the saline and L‐glutamine groups. C, Immunofluorescence staining showed the spatial relationship between HSP70^+^/GFAP^+^astrocytes and HSP70^+^/CD31^+^ endothelial cells after MCAO. Bar = 50 μm. D. TUNEL staining showed neuronal apoptosis in the 3 groups. Bar = 50 μm. N = 5 per group. Data are presented as mean ± SD. *P < 0.05, **P < 0.01, ***P < 0.001

To determine the cellular source of HSP70, we performed HSP70/NeuN, HSP70/Iba‐1, HSP70/GFAP, and HSP70/CD31 double staining. Our results showed that HSP70 was expressed in astrocytes, microglia, neurons, and endothelial cells at 72 hours after brain injury (Figure 3A), and L‐glutamine treatment increased the number of HSP70+ astrocytes and endothelial cells in the peri‐infarct area (Figure 3B). Interestingly, the detailed localization of immunofluorescent images revealed that HSP70 mainly expressed in astrocytes that wrapped around the blood vessels in the peri‐infarct area (Figure 3C). TUNEL/NeuN double staining showed that L‐glutamine significantly reduced neuronal apoptosis in the peri‐infarct area at 72 hours (Figure 3D).

### 3.4 *L‐glutamine promoted astrocytes proliferation, activated STAT3 pathway, and upregulated BDNF via HSP70 *in vivo

**Figure 4:**
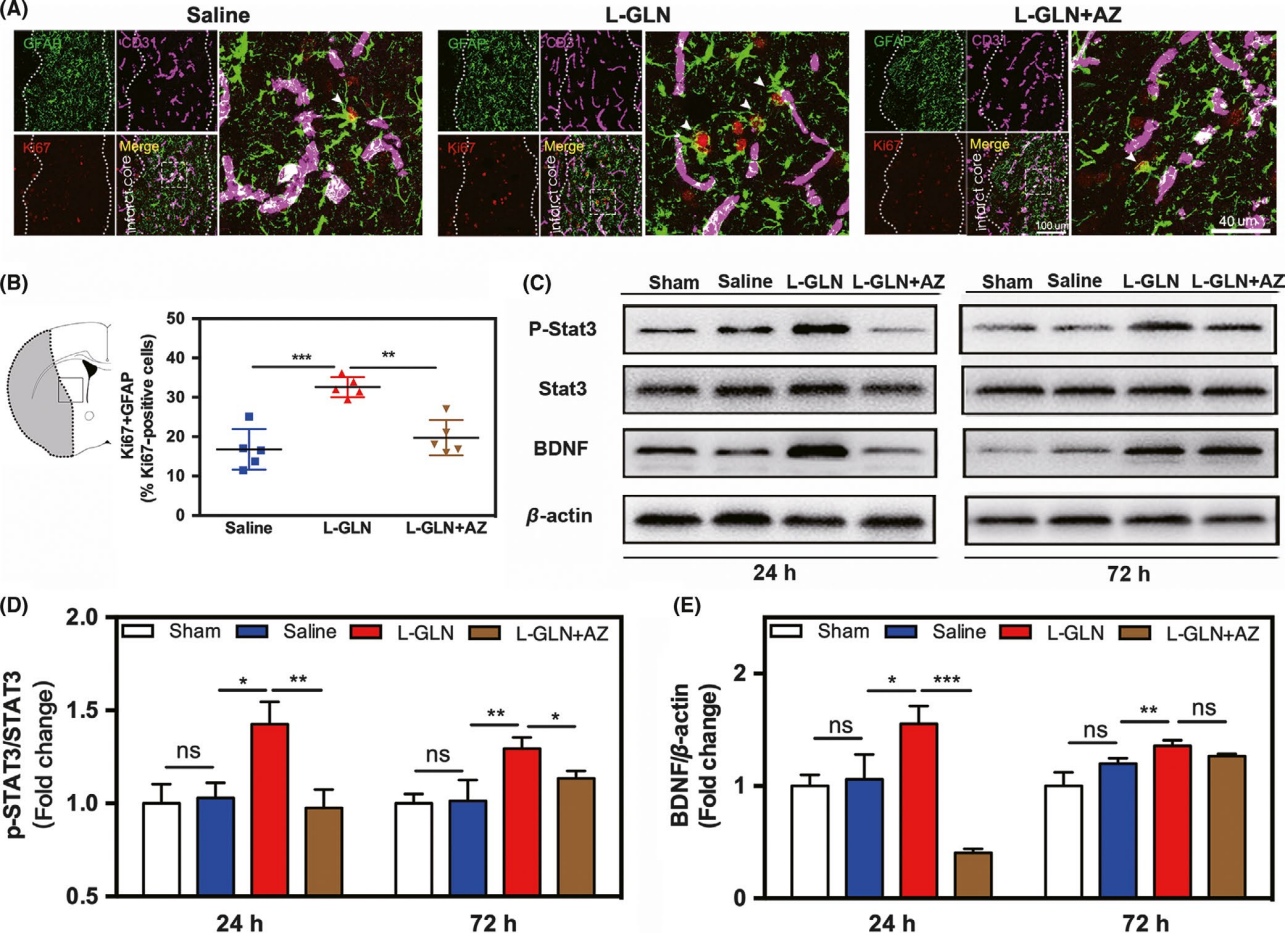
L‐glutamine promoted astrocytes proliferation, activated STAT3 pathway, and upregulated BDNF via HSP70 in vivo. A, Triple staining of Ki67 (red) and GFAP (green) and CD31 (violet) in the saline, L‐glutamine, and L‐glutamine + AZ groups. Bar = 100 μm/40 μm. B, The quantitative analysis of the number of Ki67^+^/GFAP^+^ cells. C, Western blot analysis of p‐STAT3, STAT3, and BDNF expression in the sham, saline, L‐glutamine, and L‐glutamine + AZ groups. Quantitative analysis of p‐STAT3 (D) and BDNF (E). N = 5 per group. Data are presented as mean ± SD. *P < 0.05, **P < 0.01, ***P < 0.001

To evaluate the effects of L‐glutamine on the proliferation of astrocytes and endothelial cells, immunostaining and Western blot were performed. Our results showed the number of GFAP+/Ki67+ astrocytes was increased in the lesion zone of L‐glutamine‐treated mice, while the combination of L‐glutamine with Apoptozole abolished such increase (Figure 4A,B). However, there are few CD31^+^/Ki67^+^ cells among three testing groups. Western blot analysis of the peri‐infarct tissue revealed that L‐glutamine treatment increased p‐STAT3 and BDNF expression at both 24 and 72 hours after ischemic stroke, while the combination of L‐glutamine with Apoptozole abolished these upregulations (Figure 4C‐E).

### 3.5 L‐glutamine improved the survival of astrocytes and BEND.3 cells and the conditioned medium of astrocyte promoted neuronal survival after OGD

**Figure 5:**
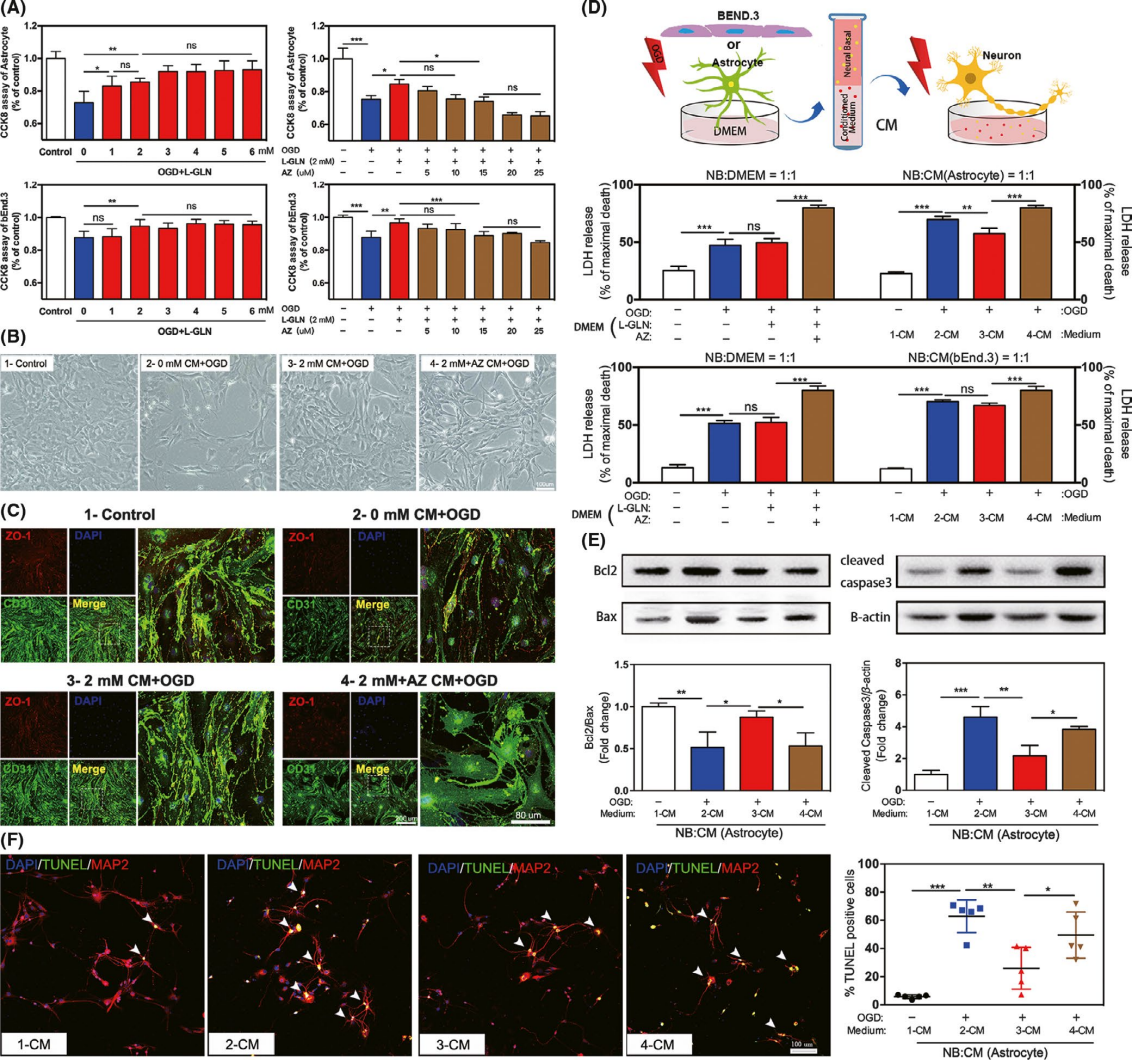
L‐glutamine promoted the proliferation of astrocytes and BEND.3 cells and the CM of astrocyte promoted neuronal survival after OGD. A, Cell viability of astrocytes and BEND.3 cells after treatment with different concentration of L‐glutamine and Apoptozole. Representative images showed the morphology of astrocyte (B) and BEND.3 cells (C) under OGD after L‐glutamine and Apoptozole treatment. Bar = 100 μm/200 μm/80 μm. D, LDH assay showed cell viability of neurons treated with conditioned medium from OGD‐treated astrocytes or BEND.3 cells. Conditioned medium from cells in normal culture (labeled as 1‐CM); conditioned medium from OGD‐treated cells was supplemented with 0 mmol/L (labeled as 2‐CM) or 2 mmol/L (labeled as 3‐CM) L‐glutamine or 2 mmol/L L‐glutamine plus 15 μmol/L Apoptozole (labeled as 4‐CM). E, Western blot analysis of BCL2/BAX and cleaved caspase 3 in neurons that treated with 1‐CM, 2‐CM, 3‐CM, and 4‐CM after OGD. F, Double staining of TUNEL (green) and MAP2 (red) of neurons in the 4 groups. Bar = 100 μm. N = 5 per group. Data are presented as mean ± SD. *P < 0.05, **P < 0.01, ***P < 0.001

CCK8 assay showed that L‐glutamine improved the survival of astrocyte and BEND.3 cells after OGD. Coadministration of Apoptozole and L‐glutamine abolished the survival effect (Figure 5A). Phase contrast imaging showed astrocytes in the L‐glutamine group after OGD (Figure 5B), and immunofluorescent double staining of CD31 and ZO‐1 indicated L‐glutamine alleviated the disruption of tight conjunction between endothelial cells (Figure 5C). The neurons were treated with conditioned media (CM) from astrocytes or BEND.3 cells to examine the effect on damaged neurons after OGD (Figure 5D). In order to balance the interference of residual drugs in CM on neurons, we used fresh medium from different groups as the control. The LDH results of neuron culture revealed that the CM from L‐glutamine‐treated astrocyte decreased the cytotoxicity of cells while no effect was detected in CM from L‐glutamine‐treated BEND.3 cells (Figure 5D). In addition, the results of Western blot showed that CM from L‐glutamine‐treated astrocyte increased the BCL2/BAX ratio and reduced expression of apoptosis‐related proteins cleaved caspase 3 as well as decreased number of apoptotic neurons (Figure 5E,F).

### 3.6 *L‐glutamine protected astrocytes from oxidative stress and increased the expression of STAT3, Nrf2, and BDNF via HSP70 *in vitro

**Figure 6:**
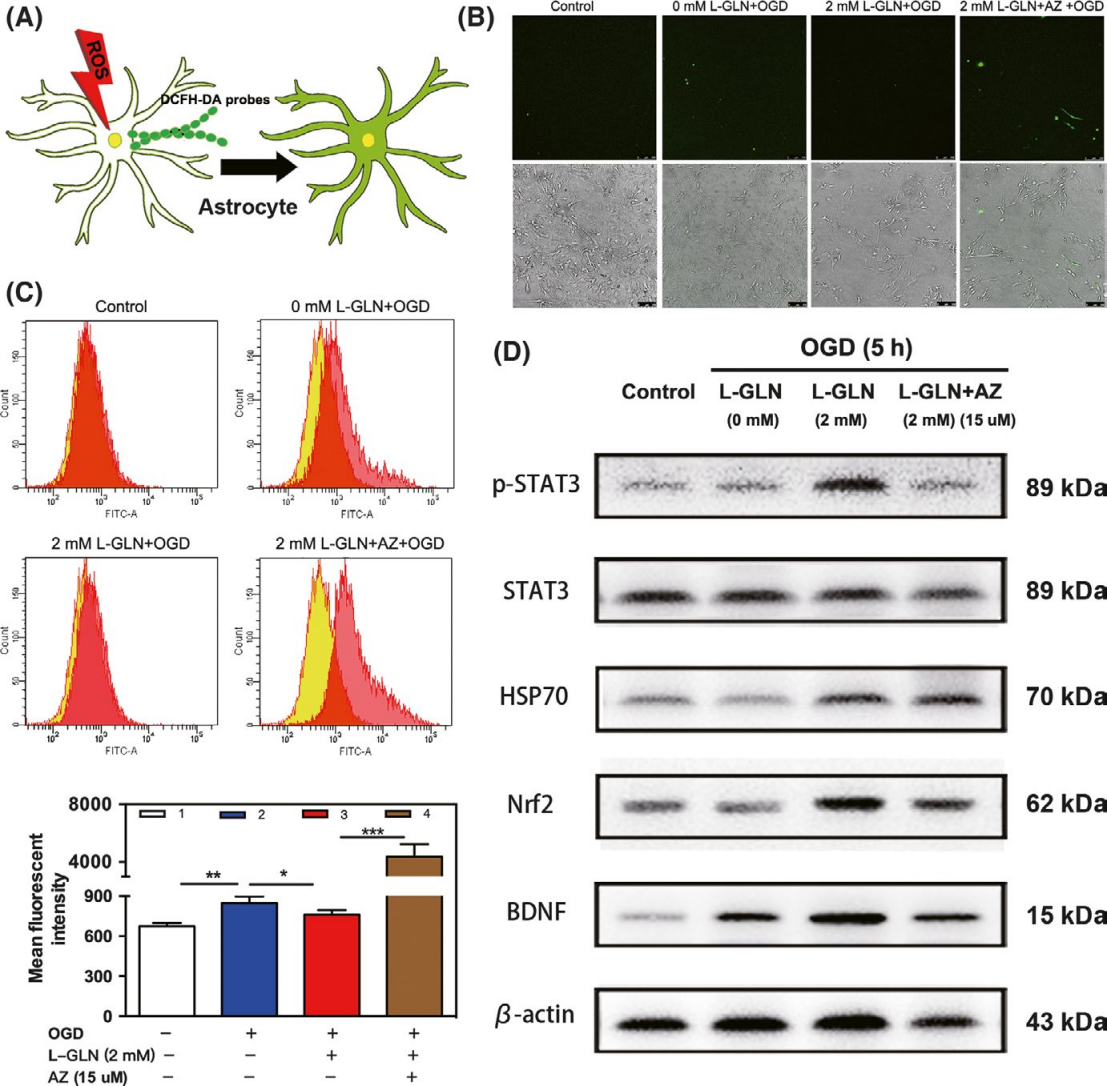
L‐glutamine protected astrocytes from oxidative stress and increased HSP70. A, DCFH‐DA probes were loaded in astrocytes to measure the intracellular ROS. Fluorescent imaging (B) and flow cytometry (C) showed the level of ROS in astrocytes that treated with DCFH‐DA. Bar = 100 μm. The overlaid histogram shows the shift of fluorescence intensity (yellow for control and red for treated samples), n = 5 per group. D, Western blot analysis of STAT3, HSP70, Nrf2, and BDNF in astrocytes cultured in 4 groups, n = 3 per group. Data are presented as mean ± SD. *P < 0.05, **P < 0.01, ***P < 0.001

To investigate whether L‐glutamine protected astrocytes from OGD‐induced injury by preventing intracellular ROS, we loaded the cells with ROS probe DCFH‐DA (Figure 6A). The results revealed that OGD increased ROS production whereas L‐glutamine treatment decreased OGD‐induced ROS accumulation, and the combination of L‐glutamine with Apoptozole abolished the effect (Figure 6B‐C). Furthermore, our Western blot results confirmed that L‐glutamine increased STAT3, HSP70, and Nrf2 expression in astrocytes. We also found that L‐glutamine treatment increased BDNF expression while such upregulation was abolished by addition of AZ (Figure 6D), indicating the effects were related to the functional activity of HSP70.

## 4 DISCUSSION

L‐glutamine plays an essential role in promoting and maintaining the function of many organs, and studies have shown that L‐glutamine enhanced HSP expression in lung injury models.^10^ HSP70 is the major stress‐inducible member of the HSP family which is expressed at low levels in nearly all intracellular compartments.^25^ Our study demonstrated that L‐glutamine treatment increased the mRNA levels of HSP32, HSP70, and HSP110 in mouse brain after stroke, while HSP90 decreased compared to the saline group (Figure S2A, Table S2). HSP70 was upregulated in response to cell stress and protected tissues and organs against brain ischemic injury. HSP70 induction initially occurs within neurons of the penumbra, and it also can be detected in endothelial and glial cells in the area adjacent to the infarct.^26^ Astrocytes and endothelial cells are less vulnerable than neurons in neurodegenerative diseases,^27^, ^28^ which is probably due to the different activity of C terminus of Hsc70‐interacting protein (CHIP) and HSP‐BP1 in different cells.^29^ It had been reported that the overexpression of HSP70 in mice improved survival of neurons and astrocytes from ischemia and ischemia‐like insults.^30^, ^31^ In our study, we revealed that L‐glutamine upregulated astrocyte‐ and EC‐derived HSP70 in the ipsilateral hemisphere, and protected neuronal survival in mice after stroke.

L‐glutamine is an indispensable nutrient for cell cycle progression through the G1 phase.^32^ L‐glutamine supplementation has been reported to promote cell proliferation and protect gut barrier.^33^, ^34^ Our results confirmed that L‐glutamine administration promoted the proliferation of astrocytes in the peri‐infarct area. Multiple experimental evidence indicates that the process of reactive astrocyte proliferation exerts a necessary beneficial function.^35^, ^36^, ^37^ Astrocyte proliferation plays an important role in regulating brain microenvironment through extracellular neurotransmitter regulation^38^ and neural growth factor secretion^39^ in the period of acute cerebral ischemia. Acting as a transcriptional factor that involved in cell survival and proliferation, STAT3 is an early trigger for astrogliosis.^40^, ^41^ BDNF is mainly secreted by astrocytes, which affect neuronal differentiation and survival. Here, we found that p‐STAT3 and BDNF levels increased in parallel to HSP70 upregulation, indicating that L‐glutamine‐induced HSP70 was involved in the proliferation of astrocytes by activating p‐STAT3 pathway and promoted the secretion of BDNF after ischemic stroke (Figure 6D).

Given that oxidative stress is a key component of ischemic stroke pathology, it is important to improve organic antioxidative effect and attenuated lipid peroxidation in stroke.^6^ In the enzyme antioxidant system, SOD and GSH are the most important antioxidants which work together to counteract oxidative stress in cells and protect brain from ROS damage.^42^ Nrf2 regulates the expression of antioxidant proteins to protect against oxidative damage.^43^, ^44^ Recent studies have indicated that L‐glutamine augments the binding of Nrf2 onto BCL2 gene promoter and protects against ischemia‐reperfusion injury in vivo by activating the Nrf2/Are signaling pathway to inhibit ROS production and reduce cell apoptosis.^45^, ^46^ Our data revealed that the expression of Nrf2 paralleled the upregulation of HSP70 induced by L‐glutamine in the peri‐infarct region at 24 hours after reperfusion, suggesting that L‐glutamine may be an activator of Nrf2 activity after ischemic stroke (Figure 6D). HSP70 modulates inflammatory responses by inhibiting the activation of the inflammatory transcription factor (NF‐κB) and prevents the formation of apoptotic bodies and subsequent caspase‐9 activation by interacting with Apaf1.^47^, ^48^ Our present findings confirmed that L‐glutamine‐induced HSP70 triggered the release of antiinflammatory cytokines (TGF‐β and IL‐10) and reduced inflammatory factors (IL‐1β and IL‐6) (Figure S1C) via NF‐κB pathway, as well as downregulated BAX/BCL2 in the ischemic penumbra to inhibit apoptosis.^48^

In our present study, we demonstrated that L‐glutamine treatment has a protective effect on cerebral ischemic injury by reducing oxidative stress, inflammatory response, and promoting astrocyte proliferation, accompanied by the upregulation of HSP70. Such beneficial effects were abolished by the coadministration of Apoptozole, indicating the central role of HSP70 in the protective effect of L‐glutamine. It is noted that L‐glutamine has been already approved by the FDA for the treatment of sickle cell disease, suggesting its clinical safety. We believe the drug possesses high potential from bench‐side to bedside for ischemic stroke.

## 5 CONCLUSION

Our work demonstrated that L‐glutamine reduced brain infarct volume and promoted neurobehavioral recovery in mice after brain ischemia, which were associated with HSP70 (Figure S3), opening a new avenue for treating ischemic stroke.

## CONFLICT OF INTEREST

The authors declare no conflicts of interest.
