# Supplementary material for: L‐glutamine protects mouse brain from ischemic injury via up‐regulating heat shock protein 70
Source: CNS Neurosci Ther. 2019 Jun 20;25(9):1030–41. doi: 10.1111/cns.13184 (PMC6698979; doi:10.1111/cns.13184)
Supplement: Supplementary file 1 [file CNS-25-1030-s001.pdf]

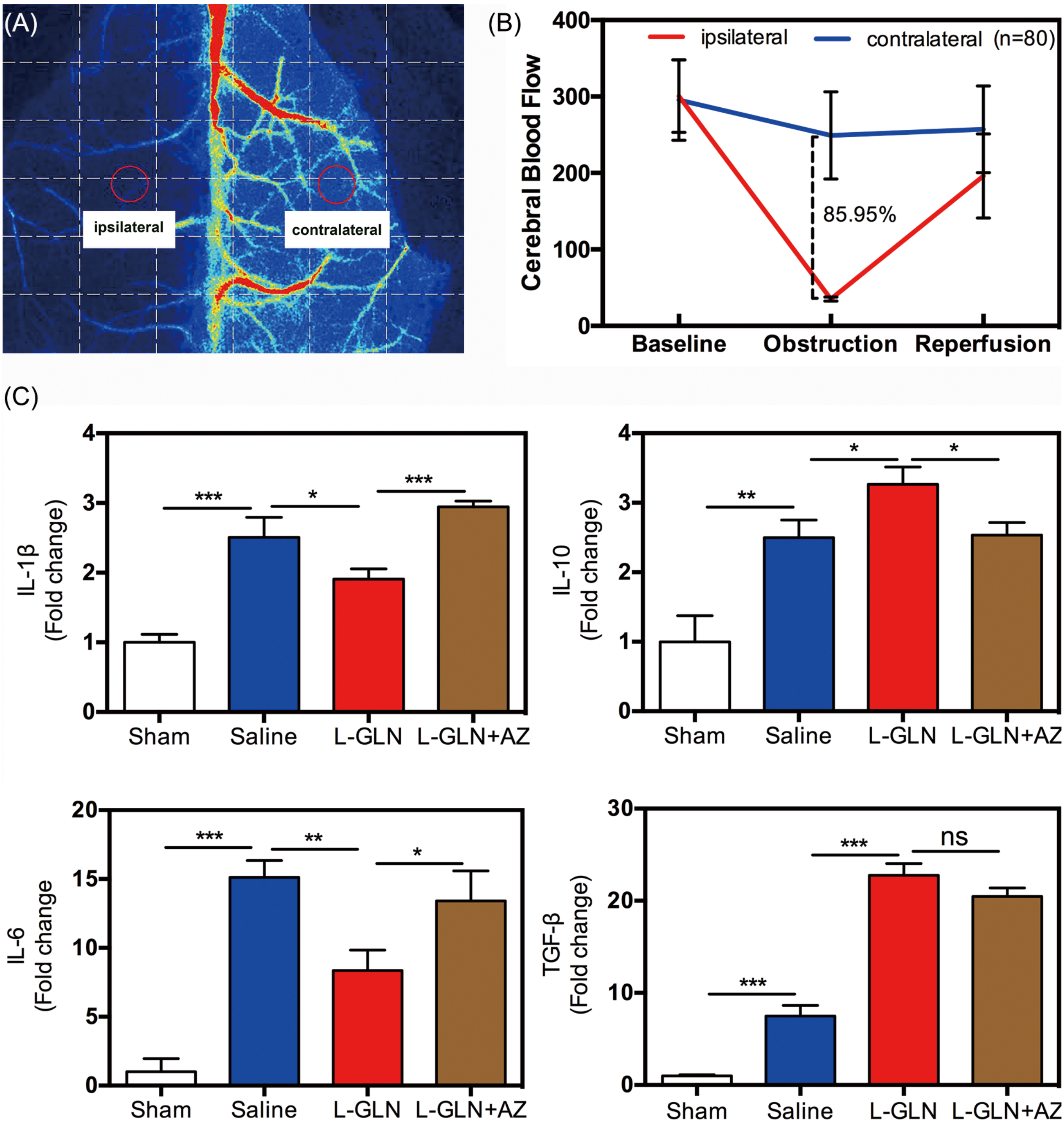

Supplemental Figure 1. Changes of blood flow before and after MCAO. A. Laser speckle imaging of cerebral blood flow after MCAO. B. Statistics of blood flow in ipsilateral and contralateral before and after reperfusion in mice. C. The mRNA level of IL-1β, IL-6, TGF-β, IL-10 at 24 hours of the 4 groups after MCAO.

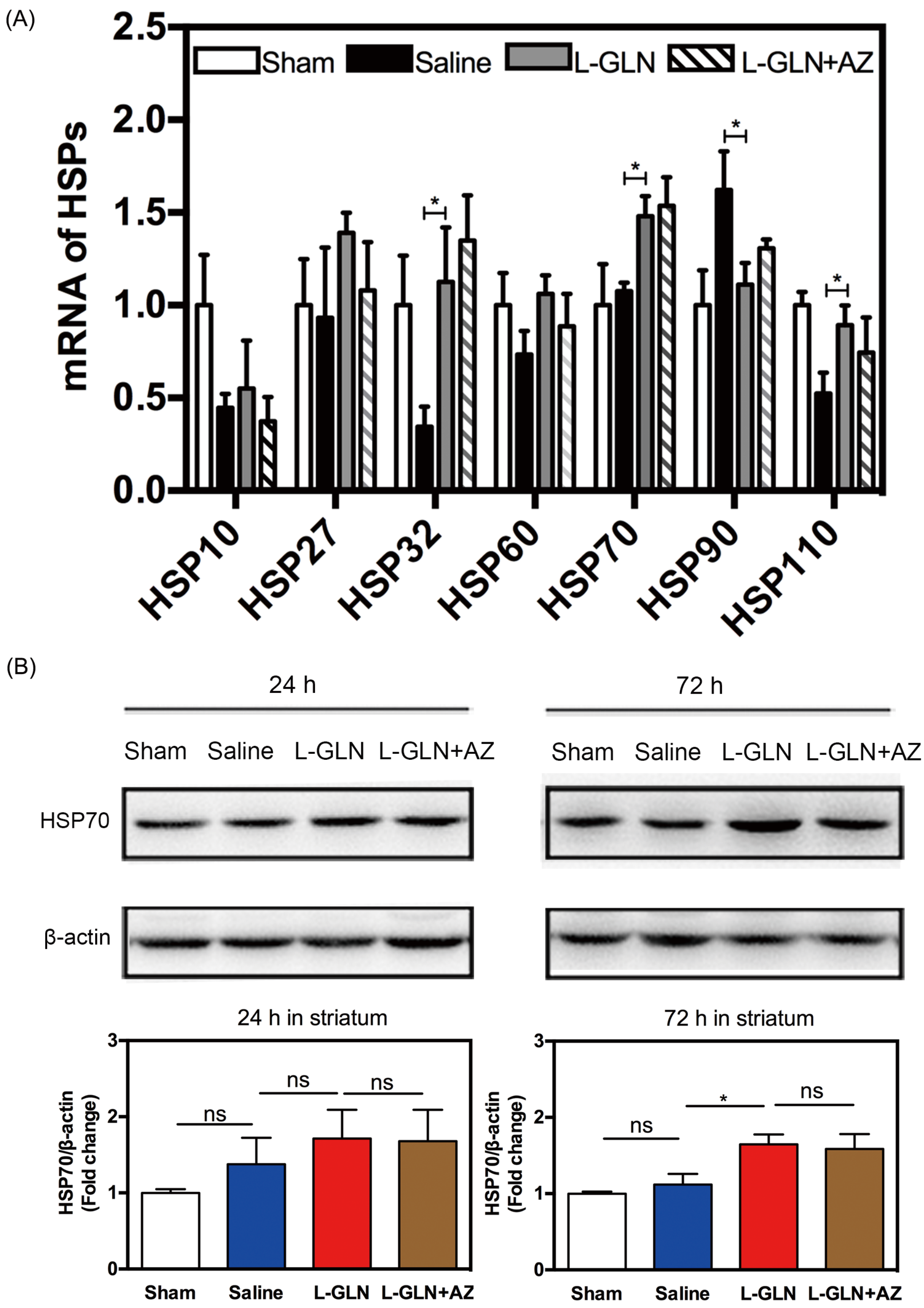

Supplemental Figure 2. The mRNA level of HSPs in mice that treated with L-GLN at 72 hours after stroke. A. The mRNA level of HSPs in sham, saline treated, L-GLN treated and L-GLN+AZ treated mice. B. Western blot analysis of HSP70 from the 4 groups at 24 and 72 hours in striatum. N=5 per group. Data are presented as mean ± SD. *$p < 0.05$, **$p < 0.01$, ***$p < 0.001$.

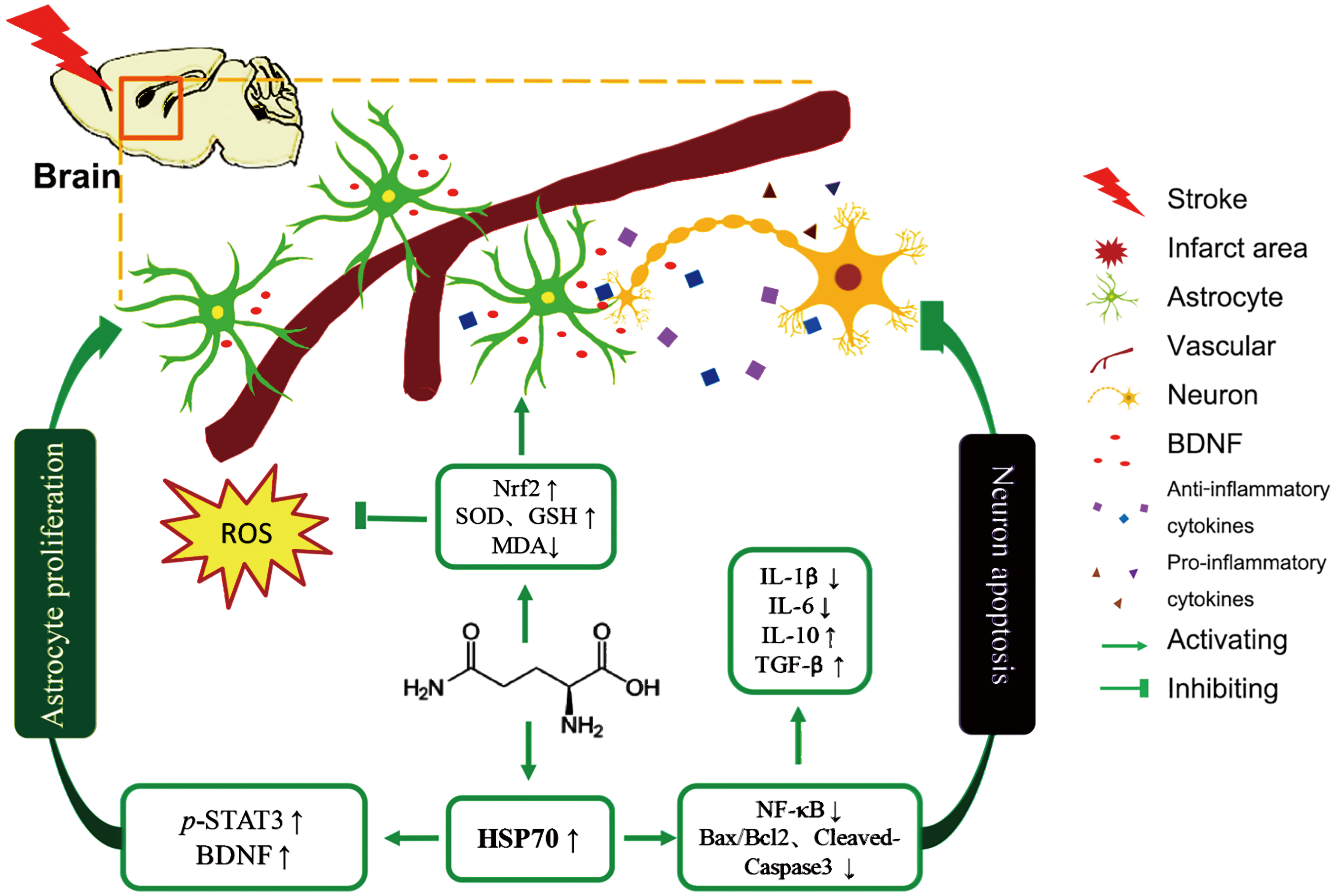

Supplemental Figure 3. Proposed mechanisms underlying the action of L-glutamine in acute cerebral ischemic stroke. L-glutamine induces HSP70 up-regulation, further enhances astrogliosis by activating STAT3 and BDNF expression, and protects neurons from damage via anti-apoptosis, anti-inflammation and anti-oxidation pathway.

Supplemental Table 1

Test	Aim	Process
mNSS	To assess the neurological deficit (scores range from 0 to 14, with 0 representing normal and 14 representing the most severe)	To test the motor function, the animal is raised by the tail to examine its forelimb flexion (0-3) and its gait on the flat surface was scored (0-3). For the balance test, animal is placed on a beam, and a score ranging from 0 to 6 is given according to whether the mouse could keep balance, limbs fall or walk through the beam. The pinna and corneal reflex are examined in reflexes absent test (0-2).
EBST	To determine the balance ability of animals	The animal is held approximately 10 cm from the test platform of its tail and is fixed in the vertical axis, defined as no more than 10 ° to either the left or the right side. A swing is recorded whenever the animal moved its head out of the vertical axis to either side. Swings are recorded using a hand counter. The total number of swings made to each side is divided by the over all total number of swings made to both sides to get percentages of left and right swings.
Rotor-Rod	To assess sensorimotor coordination and motor learning in rodent models of CNS disorders	Mice are placed on a rotating rod with either constant rotation or a steady acceleration; At least 3 days of training are needed to ensure that all mice have learned the task to the same degree. During training, mice learn to balance on a stationary rod, then on a rod constantly rotating at 20 rpm. In the formal rotation test, the mice are placed on a rod which accelerates to and then constantly rotates at 40 rpm. The latency to fall is recorded. A trial is complete when the animal falls or the time period ends.
Hanging wire tests	To assess global “subacute” muscle function and coordination over time	Mice are subjected to 180 second lasting hanging test, during which a “falling” and “reaching” scores are recorded. When a mouse falls or reaches one of the sides of the wires, the “falling” score or “reaching” score are diminished or increased by one respectively. A Kaplan-Meier-like curve can be created afterwards. For this test a 55 cm long wire was used which remained constant between different labs.

Supplemental Table 2

Gene	Forward primer (5′–3′)	Reverse primer (5′–3′)
HSP10	GTATTGGTTGAAAGGAGTG	AAGTTGGAGATAAAGTTCTTCT
HSP27	CAAACGGGTCATTGCCATTA	AAAGCGCAGCCGAGCCCAGC
HSP32	TCTGGAATGGAGGGAGATAC	TGAACTTTGAAACCAGCAG
HSP60	TAATGCTCATCGGAAGCCAT	ACGTTGACGGAGAAGCTCTA
HSP70	TATCAGTGTTCCAGTAGCCTG	TTTACTCTTTTGTACTTAAA
HSP90	AAGAAGGTTGAAAAGGTGGT	CAAGCCCTCAGAGACAACTC
HSP110	ATGAGTTCTAACAGCACGGACC	CAAGGATGTCTCTGGGAAGATGA
IL-1β	CGCAGCAGCACATCAACAAGAGC	TGTCCTCATCCTGGAAGGTCCACG
IL-6	TGATGCACTTGCAGAAAACAA	GGTCTTGGTCCTTAGCCACTC
TGF-β	CACCGGAGAGCCCTGGATA	TGTACAGCTGCCGCACACA
IL-10	GCGCTGTCATCGATTTCTCC	TGGCCTTGTAGACACCTTGG
GADPH	AAATGGTGAAGGTCGGTGTG	AGGTCAATGAAGGGGTCGTT
